# Dietary muramidase leads to the downregulation of peptidoglycan biosynthesis and to caecal microbial modulation in laying hens

**DOI:** 10.1186/s42523-025-00506-9

**Published:** 2026-01-20

**Authors:** Salvatore Galgano, Murtala Umar Faruk, Irene Eising, Jos G. M. Houdijk, Farina Khattak

**Affiliations:** 1 Monogastric Science Research Centre, School of Veterinary Medicine and Biosciences, Scotland’s Rural College (SRUC), Edinburgh, UK; 2 dsm-firmenich Nutritional Products Ltd., Kaiseraugst, Switzerland

**Keywords:** Laying hen, Lysozyme, Metagenomics, Microbiome, Muramidase, Peptidoglycan, Short read

## Abstract

**Background:**

In-feed muramidase enzyme has been linked to numerous advantages in several animal species. In the past years, muramidase has been shown to be effective in hydrolyzing peptidoglycan fragments, especially at small-intestine level in broilers, and to improve digestibility and performance. Moreover, previous studies also showed a possible anti-inflammatory effect of some secondary metabolites derived from the hydrolysis of peptidoglycan. Although a major effort has been carried out to unravel the in vivo mechanism of action of muramidase, there is currently little information on its metabolic interactions in laying hens, especially considering the fundamental differences with broilers in terms of microbiota and host genetics. Therefore, we conducted a 20-week study, testing five different levels of inclusion of muramidase, from 0 mg/kg to 600 mg/kg. We analyzed dry matter and nitrogen digestibility, apparent metabolizable energy, body weight gain, caecal microbiota and microbiome.

**Results:**

The intervention with muramidase (Balancius^®^, DSM Nutritional Products Ltd., Basel, Switzerland) led to a drop in α-diversity (Shannon index; *P* < 0.05) and to microbial composition changes, with a decrease in *Lactobacillus* and an increase in *Collinsella*, amongst others (*Q* < 0.05), at all the muramidase concentrations compared to 0 mg/kg. In parallel, we found that muramidase led to an increased protein digestibility as revealed by the increased nitrogen retention, together with a dose-dependent amelioration of body weight, dry matter digestibility and metabolizable energy (*P* < 0.05). At functional gene level, we observed a net decrease in the microbial potential to metabolize amino acids, likely as a direct consequence of the lower amino acid availability at caecal level, as linked to the increased nitrogen retention. Moreover, muramidase also led to a decreased microbial functional potential to synthesize peptidoglycan.

**Conclusion:**

This study is the first to investigate the effects of dietary muramidase supplementation on nutrient digestibility and metagenomics in laying hens. Our findings align perfectly with the previous studies in broilers, especially in terms of increased protein digestibility. Moreover, for the first time, a direct correlation between the observed phenotype and both microbiota and microbiome has allowed us to gain further insights into the mechanism of action of muramidase in laying hens.

**Supplementary Information:**

The online version contains supplementary material available at 10.1186/s42523-025-00506-9.

## Background

The bidirectional interactions between gut microbiota and host regulate a multitude of functions, ultimately affecting the host phenotype [1, 2]. Hence, gut microbiota manipulations could possibly lead to ameliorated traits such as performance, nutrient digestibility and absorption [3], or gut functionality improvement [4]. Peptidoglycan (PGN) is the main component of the bacterial cell wall, especially for Gram positive bacteria [5], whilst muramidases (EC 3.2.1.17) of the class hydrolases, also known as lysozymes, catalyze the hydrolysis of the PGN at β-1, 4 glycosidic linkages level, between N-acetylmuramic acid and N-acetyl-D-glucosamine residues [6, 7]. Although genetically conserved, at least three different types of muramidase have been reported; c-type (common or chicken), g-type (goose), and i-type (invertebrate), with principal differences in biochemical/enzymatic properties and amino acid sequence [8].

Importantly, beyond its primary enzymatic role in PGN degradation, muramidase can also lead to immune modulation [9]. This is particularly significant in light of the three muramidase resistance mechanisms; PGN modification, charge and integrity alterations of the cell wall, and the synthesis of muramidase inhibitors [9]. Linked to this, antimicrobial resistance is a worldwide problem derived from the acquired or innate bacterial resistance to common antimicrobials, including antibiotics [10], and mainly caused by their misuse [11]. This is of particular relevance to livestock in view of the past application of antimicrobials as growth promoters (AGP) consisting of low-dose administration of antibiotics correlated with a significant improvement in performance [12]. After the AGP ban, several studies investigated different approaches to improve health and nutrient utilization through microbiota modulation, such as dietary interventions, probiotics, and prebiotics [13–16]. In parallel, numerous authors described the positive effects of egg-white muramidase in farm animals, such as pigs and poultry, in terms of increased performance and immunity/gut health markers [17–19]. More recently, a muramidase from *Acremonium alcalophilum* (AcM) was shown to be safe in poultry [20, 21], and its efficacy towards performance, meat yield, and welfare was confirmed in both broilers and turkeys [22, 23]. A recent investigation of its mechanism of action showed that AcM does not exhibit direct antimicrobial activity as it does not target the PGN of live bacteria but rather luminal cell-free PGN fragments, whilst it is able to promote anti-inflammatory pathways through nucleotide-binding oligomerization domain-containing protein 2 receptor activation [24]. In addition, the administration of AcM was correlated with systemic reduction of hypoxanthine, histidine, and uracil, but increased pyruvate, 2-oxoglutarate, and glucose in connection to a decreased caecal α-diversity and improved performance in broilers [25].

To the best of our knowledge, no information is currently available on the effects of generic muramidase or AcM in laying hens and associated microbiota. Although taxonomically belonging to the same species (*Gallus gallus domesticus*), years of selective breeding led to many genetic differences between broilers and layers [26]. Moreover, important differences are also to be observed in their microbiota, which not only present different composition and functional potential [27], but due to the different lengths of the production cycle, it could result in being more established in layers. We carried out a 20-week study with a total of 900 hens to assess the effects of four dietary inclusion levels of AcM on nutrient digestibility (i.e., nitrogen retention, dry matter digestibility and apparent metabolizable energy) and on the caecal microbiota and microbiome. Our analyses allowed us to establish a parallelism around the bi-directional interactions between host and bacteria, ultimately informing on the possible cause-effect relationship behind the observed phenotype.

## Methods

### Animal study

The study was approved by the SRUC animal welfare and ethical review body (AEX 2022-002 POU) and carried out at the SRUC Allermuir Avian and Innovation Skill Centre (AISC). A total of 900 Lohmann brown hens, aged 22 week old, obtained from a commercial farm (Noble Foods, Kirkcaldy, United Kingdom), were housed in 60 enriched colony cages. Each cage had a 750 cm^2^ and 11.48 cm feed trough space per bird. Each cage was equipped with a nest, scratch mat, perches, and hanging nylon ropes for enrichment. The hens were allocated to 5 treatments where a 150 mg/kg increasing concentration of AcM (Balancius^®^, DSM Nutritional Products Ltd., Basel, Switzerland), was added to the feed at the mill (Target feeds, Whitchurch, United Kingdom). Therefore, the corresponding treatments were T1 with 0 mg/kg, T2 with 150 mg/kg, T3 with 300 mg/kg, T4 with 450 mg/kg, and T5 with 600 mg/kg of AcM. Each of the treatments had 12 replicate cages with 15 hens each. The hens were allocated to cages to ensure that the initial body weight of hens was similar across treatments. The hens were housed in an environmentally control house. The temperature and relative humidity were approximately 20 °C and 65%, respectively. Hens had *ad libitum* access to feed and water for the duration of the experiment. Cage-level body weight was measured at the start and end of the study, allowing the calculation of the body weight gain (BWG) whereas feed intake was calculated fortnightly. Maize, barley, wheat, and soyabean based diet (T1) was manufactured at Target feeds Ltd, Whitchurch, United Kingdom (see Additional file 1 for the detailed diet composition) and then additional 4 treatments (T2, T3, T4 & T5) were generated by the addition of AcM according to the inclusion levels stated above. The AcM enzyme used in this study is same as the one previously described [28] and marketed under the trade name of Balancius^TM^ (dsm-firmenich animal nutrition and health, Wurmisweg, 576, 4303, Kaiseraugst, Switzerland).The gene coding for this AcM is from the fungus *Acremonium alcalophilum* (strain 114.92), and its activity is expressed in MUR units LSU(F).

Titanium dioxide (TiO_2_) was added as an inert marker to determine digestibility.

### End of study sample collection

At the end of the study, at experimental day 140, an aliquot of 25 g of excreta sample was collected from each cage and freeze-dried (James Hutton Institute, Aberdeen, UK). Both feed and excreta samples were analyzed for titanium dioxide (TiO_2_), nitrogen (N), and gross energy (GE) content at Sciantec Analytical Laboratory, UK, to determine nitrogen retention (NR), dry matter digestibility (DMD) and apparent metabolizable energy (AME). GE was analyzed through an isoperibol calorimeter system using benzoic acid as an internal standard (model 1261, Parr Instruments, Moline, Illinois, USA) at Pemberton Analytical Services (Shropshire, UK). Consequently, AME, DMD and NR were calculated using Eqs. 1, 2 and 3, shown below:

1 $$\:AME\left(kcal/kg\right)={GE}_{\left(feed\right)}-\left[{GE}_{\left(excreta\right)}\cdot\:\left(\frac{{TiO}_{2\left(feed\right)}}{{TiO}_{2\left(excreta\right)}}\right)\right]$$

2 $$\:DMD\left(\mathrm{\%}\right)=1-\left(\frac{{TiO}_{2\left(feed\right)}}{{TiO}_{2\left(excreta\right)}}\right)$$

3 $$\:NR\left(\mathrm{\%}\right)=100\cdot\:\left[DMD\cdot\:\left(\frac{{N}_{\left(excreta\right)}}{{N}_{\left(feed\right)}}\right)\right]$$

All experimental diets were analyzed for dry matter (DM), oil A (i.e., ether extraction targeting the analysis of free lipids), crude protein, crude fiber, and ash at Sciantec Analytical Services Ltd. (Cawood, UK) using standard protocols based upon Commission Regulation (EC) No. 152/2009.

Finally, one bird per cage was humanely culled, and a 0.25 g pooled caecal sample aliquot was collected into PowerBead Pro Tube from the QIAsymphony PowerFecal Pro DNA Kit (Cat. No. 938036, QIAGEN, Hilden, Germany), and transferred to − 80 °C until DNA isolation. This was performed at the SRUC Biomarkers Lab (Edinburgh, UK) following manufacturer instructions on QIAsymphony SP (Cat. No. 9001297, QIAGEN, Hilden, Germany), and adding 4 µl of RNase A per sample (Cat. No. 19101, QIAGEN, Hilden, Germany) after homogenization of the PowerBead Pro Tubes in a FastPrep-24™ 5G homogenizer (MP Biomedicals, Santa Ana, CA, USA) for 55 s at 5.5 m/s.

### Metagenomic sequencing and bioinformatic analysis

Library preparation and sequencing was carried out following a previously described protocol [29]. In brief, genomic DNA was used to carry out quantitative and qualitative control, library preparation and shotgun metagenomic sequencing on NovaSeq X Plus (Illumina, San Diego, California, United States) of paired-end 150 bp fragments with a target output of 6 Gb of raw data per sample.

The fastq reads were processed with Kneaddata, which integrated FastQC [30], Trimmomatic [31] and Bowtie2 [32], allowing quality control and decontamination via alignment to the Rhode Island red laying hen genome (https://www.ncbi.nlm.nih.gov/datasets/genome/GCA_024652985.1/) as the assembled Lohmann Brown genome was not available at the time of writing. Taxonomy was assigned using the MetaPHlAn V3.1 pipeline [33] and the version Oct22-202212 of the CHOCOPhlAn (SGB) database, outputting total sum scaling (TSS) normalized taxonomy tables. Finally, microbial functional profiling was carried out using HUMAnN 3.5 [34], through which the gene families were grouped into one feature table, normalized into copies per million (CPM), and further grouped in Kyoto Encyclopedia of Genes and Genomes (KEGG) orthologs [35–37], and pathways. Both α-diversity (richness and Shannon index) and β-diversity (Jaccard distance and Bray-Curtis dissimilarity) were carried out in QIIME2 [38] after importing both the taxonomy and functional gene feature tables as “.qza” artifacts via using the q2-sapienns plugin of the shotgun distribution (v2024-5). MelonnPan [39] was used to predict the microbial metabolic profile based on the gene families extracted from HUMAnN, therefore generating a table of predicted microbial metabolites, based on the presence of the relative encoding genes.

The R package MaAsLin2 [40] was used to carry out the multivariable associations, thanks to which the eventual correlations between metagenomic data and treatment or phenotype were assessed, whilst considering the false discovery rate. Venn diagrams were computed with the package ggvenn [41] and exported using the package gplots [42] in R. Finally, β-diversity distance matrixes were analyzed using PERMANOVA in QIIME2, whilst the visualization of both α- and β-diversity was performed via integrating the packages QIIME2R [43] and ggplot2 [44].

### Statistical analysis

The R v4.3.3 [45] package *lme4* [46] was used to carry out the linear mixed model (LMM) with treatment (or test variable) as fixed effect and block as random effect, thanks to which phenotypical and α-diversity data were analysed. The type III ANOVA analysis via Satterthwaite’s degrees of freedom was performed using the R package *lmerTest* [47] to assess the significance of the main effects, whereas significant relationships were further explored via estimated marginal means analysis using the package emmeans V1.11.1 [48], thus, a Tukey-adjusted contrasts pair wise comparison was also carried out. Finally, orthogonal polynomial contrasts were assessed with emmeans. BWG, NR and AME were log_10_ transformed pre-analysis. The taxonomical features (i.e., phyla and genera) were analyzed via carrying out a linear mixed model [46] on all the features in the data set, whilst taking into account the false discovery rate (type I error) under repeated testing through Benjamini & Hochberg correction [49], of the P-values calculated via type III ANOVA using the Satterthwaite’s method [47]. Therefore, only the features associated with both a *P* and a *Q* value < 0.05 were considered significant.

## Results

### Treatment driven phenotype

**Fig. 1:**
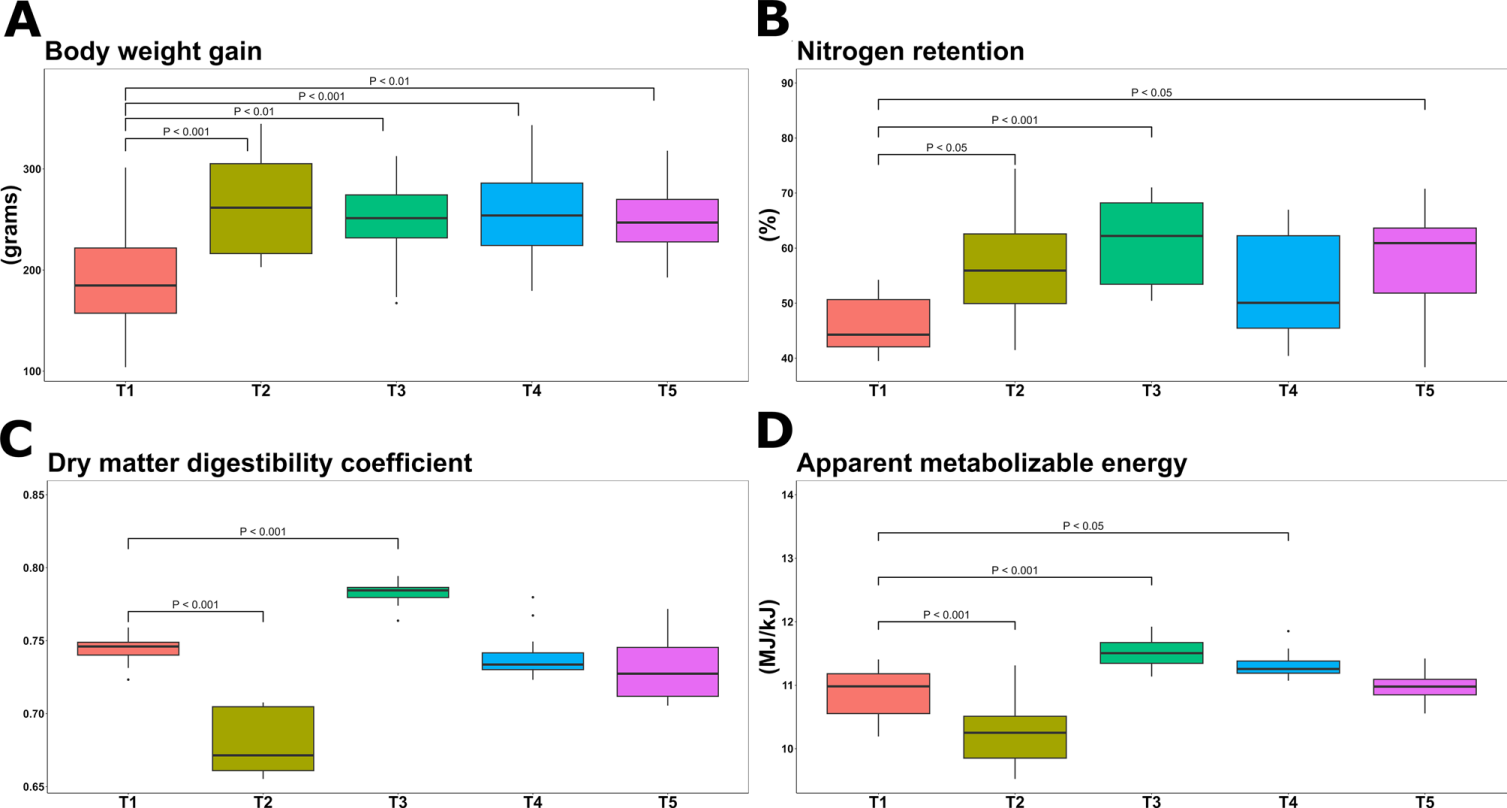
The digestibility phenotype was significantly affected by the different AcM levels of inclusion

As depicted in Fig. 1A, AcM significantly affected cumulative BWG (F_(4,44)_ = 5.3; *P* < 0.01), with increased values in T2 (265.17 g ± 54.25 g), T3 (249.33 g ± 46.1 g,), T4 (255.84 g ± 48.59 g) and T5 (252.00 g ± 42.50 g) compared to T1 (190.90 g ± 54.43 g). A significant linear relationship was found (*P* < 0.01), together with a significant negative quadratic effect (*P* < 0.01), indicating a concave relationship between treatment concentration and BWG, thus suggesting the presence of an optimal AcM concentration at T3, beyond which the BWG values appeared to decrease.

Similarly, NR was also significantly influenced by the treatment (F_(4,55)_ = 5.3; *P* < 0.01, Fig. 1B), with a significant increase in T2, T3, and T5 (56.94% ± 9.61%, 60.99% ± 8.1%, and 57.42% ± 9.82%; *P* < 0.01). Moreover, in this case the polynomial contrast revealed a significant positive linear effect (*P* < 0.05), indicating a generic increase in NR, proportional to the AcM administration. The quadratic model showed a significant negative coefficient (*P* < 0.01), likely linked to the NR pick correspondent to T3 as discussed above, whilst the cubic model showed also a positive significant coefficient (*P* < 0.05), indicating possible inflection points at increasing AcM levels.

In parallel, DMD (F_(4,44)_ = 58; *P* < 0.001, Fig. 1C) and AME (F_(4,44)_ = 25.8; *P* < 0.001, Fig. 1D) were also significantly correlated to the intervention. DMD and AME were highest in T3 (0.78 ± 0.01 and 11.53 MJ/kJ ± 0.24 MJ/kJ; *P* < 0.001, respectively), while T2 exhibited a significant decrease of both metrics (0.68 ± 0.02 and 10.22 MJ/kJ ± 0.51 MJ/kJ; *P* < 0.001) compared to T1 (0.74 ± 0.01 and 10.87 MJ/kJ ± 0.38 MJ/kJ for DMD and AME, respectively). Moreover, the polynomial contrast analysis indicated a significant linear (*P* < 0.05), cubic (*P* < 0.001) and quartic (*P* < 0.001) effects in the relationship between treatment and DMD, whilst the negative quadratic trend approached significance (*P* = 0.086), suggesting a highly non-linear relationship characterized by alternating increase and decrease of DMD across the different AcM levels with a pick on T3. Finally, the polynomial contrast revealed a significant linear (*P* < 0.05), quadratic (*P* < 0.05), cubic (*P* < 0.001) and quartic (*P* < 0.001) trends between the intervention and AME indicating multiple directional changes in the concentration of AME across the different treatment groups, but also suggesting a concave inverted-U pattern with a pick on T3.

**Fig. 2:**
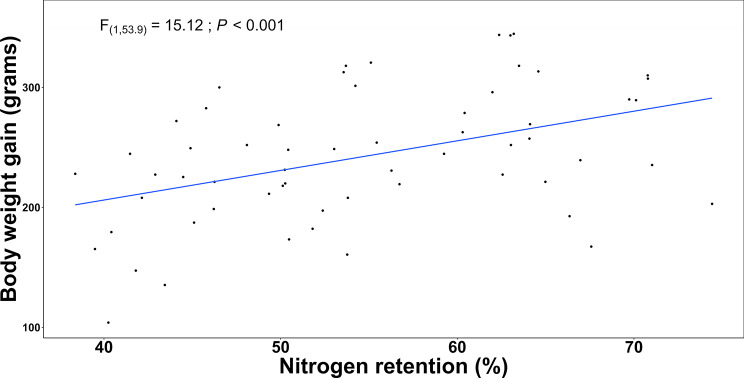
The linear mixed model revealed a significant direct relationship between BWG and NR, with the former increasing as a function of the latter

The increase in BWG was not attributed to increased FI. However, the LMM fitted with BWG as the dependent variable and NR, DMD, or AME as independent variables revealed a significant positive correlation between BWG and NR (F_(1,53.9)_ = 15.12; *P* < 0.001, Fig. 2).

### Muramidase-driven modulation of the caecal microbiota

**Fig. 3:**
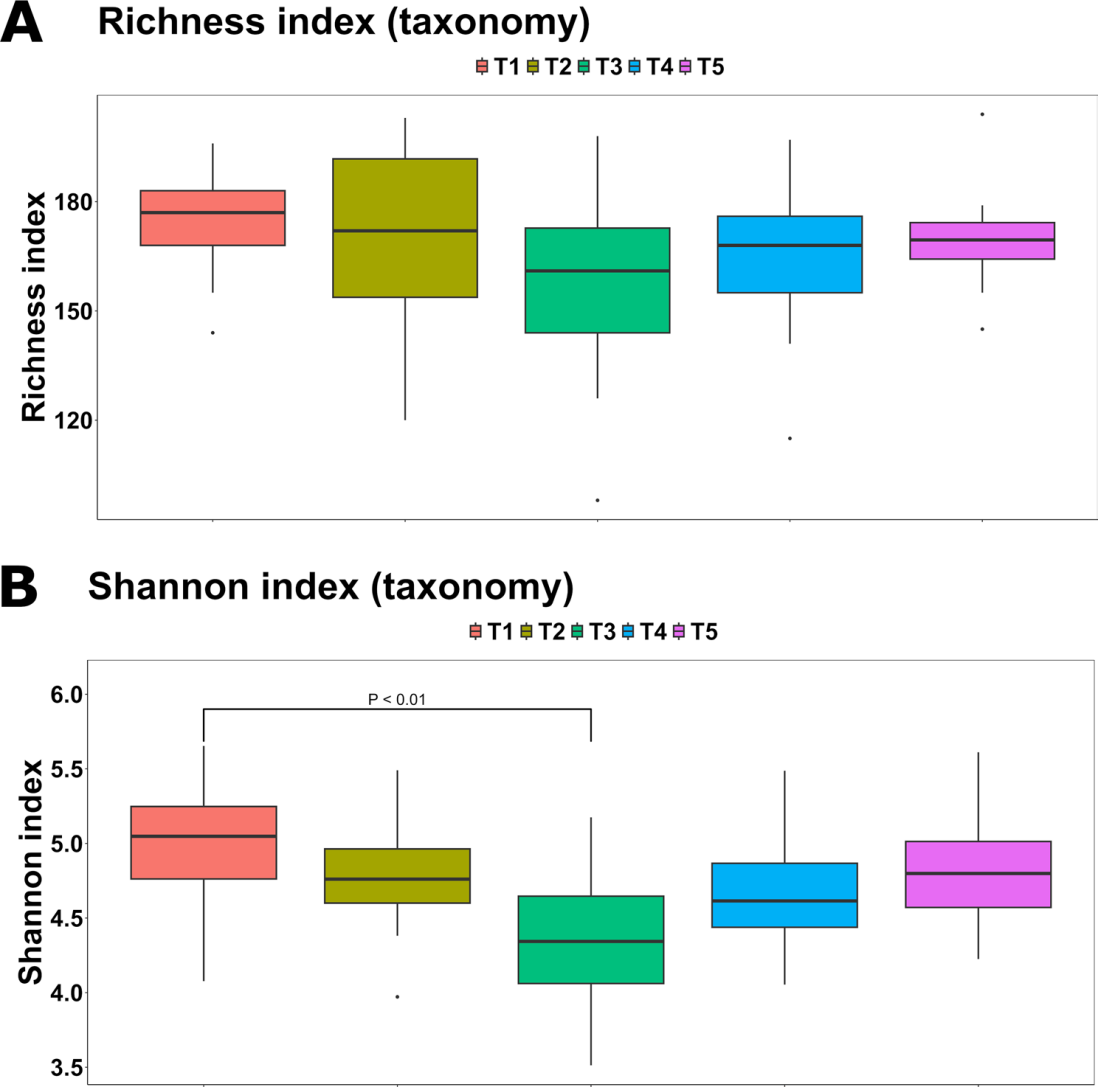
Richness (**A**) and Shannon diversity (**B**) indexes measured throughout the experimental groups. T1: 0 mg/kg; T2: 150 mg/kg; T3: 300 mg/kg; T4: 450 mg/kg; T5: 600 mg/kg (*n* = 12 cages per treatment)

**Fig. 4:**
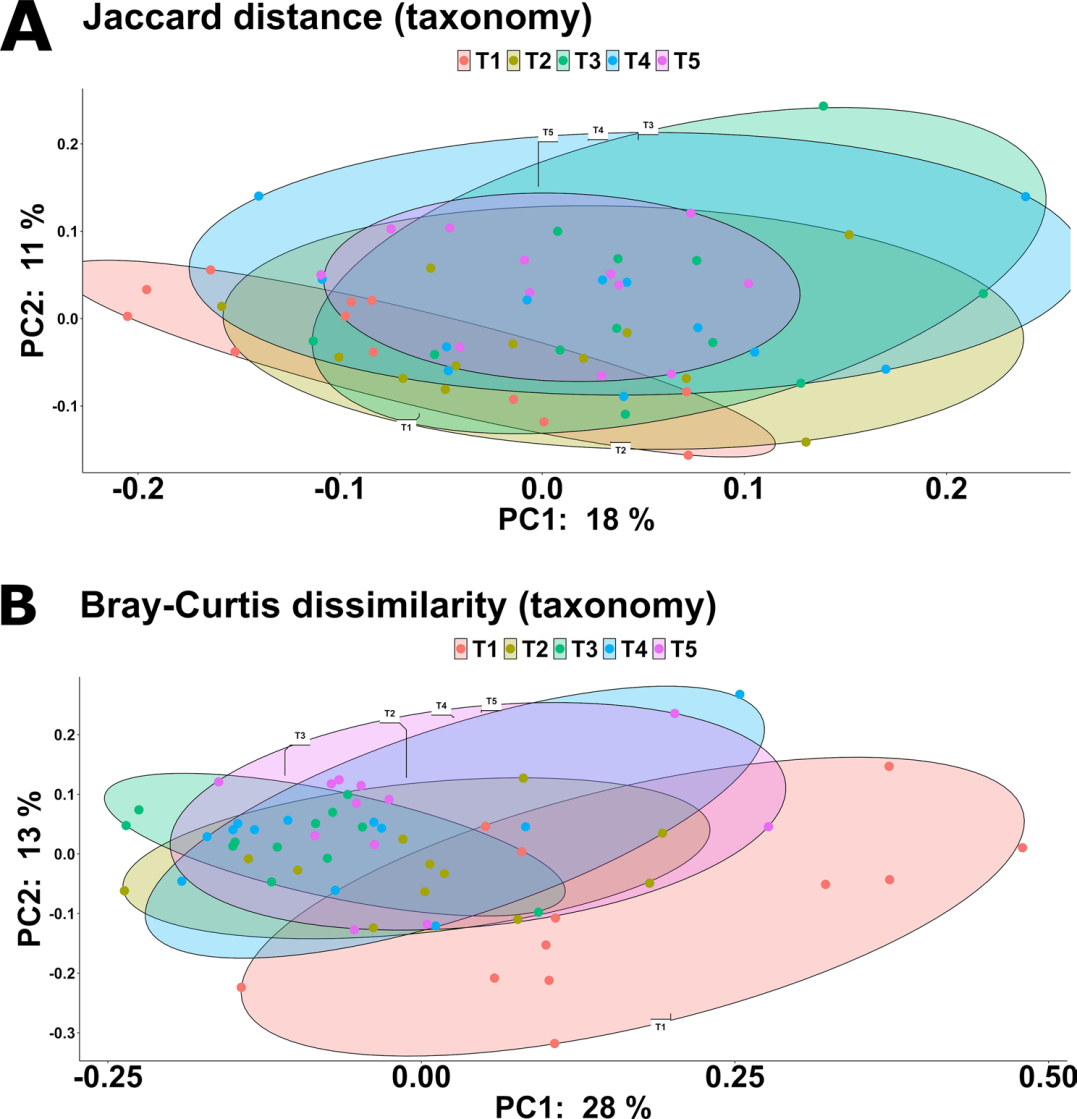
Jaccard distance (**A**) and Bray-Curtis dissimilarity (**B**) shown via principal coordinate analysis (PCoA) plot. T1: 0 mg/kg; T2: 150 mg/kg; T3: 300 mg/kg; T4: 450 mg/kg; T5: 600 mg/kg (*n* = 12 cages per treatment)

Richness did not change across the treatments (Fig. 3A); however, it was somewhat negatively correlated to NR (F_(1,53.5)_ = 3.87; *P* = 0.054), DM (F_(1,50.7)_ = 2.99; *P* = 0.090) and the log_10_ transformed BWG (F_(1,55.0)_ = 3.00; *P* = 0.089). In contrast, the Shannon diversity index was not associated with digestibility markers but was significantly correlated to the treatment (F_(4,44.0)_ = 4.15; *P* < 0.01). Notably, a reduction was observed in T3 (4.33 ± 0.45) compared to T1 (4.95 ± 0.47; *P* < 0.001), with a significant quadratic trend between the Shannon index and the different AcM levels (*P* < 0.01, Fig. 3B). In terms of β-diversity, all the treatments plotted separately from T1 (*Q* < 0.01), and T5 was significantly distant from T2 and T3 both when measuring the Jaccard distance (Fig. 4A) and the Bray-Curtis dissimilarity (Fig. 4B), whilst the Bray-Curtis distance between T3 and all the remaining treatments was also significant (*Q* < 0.05).

**Fig. 5:**
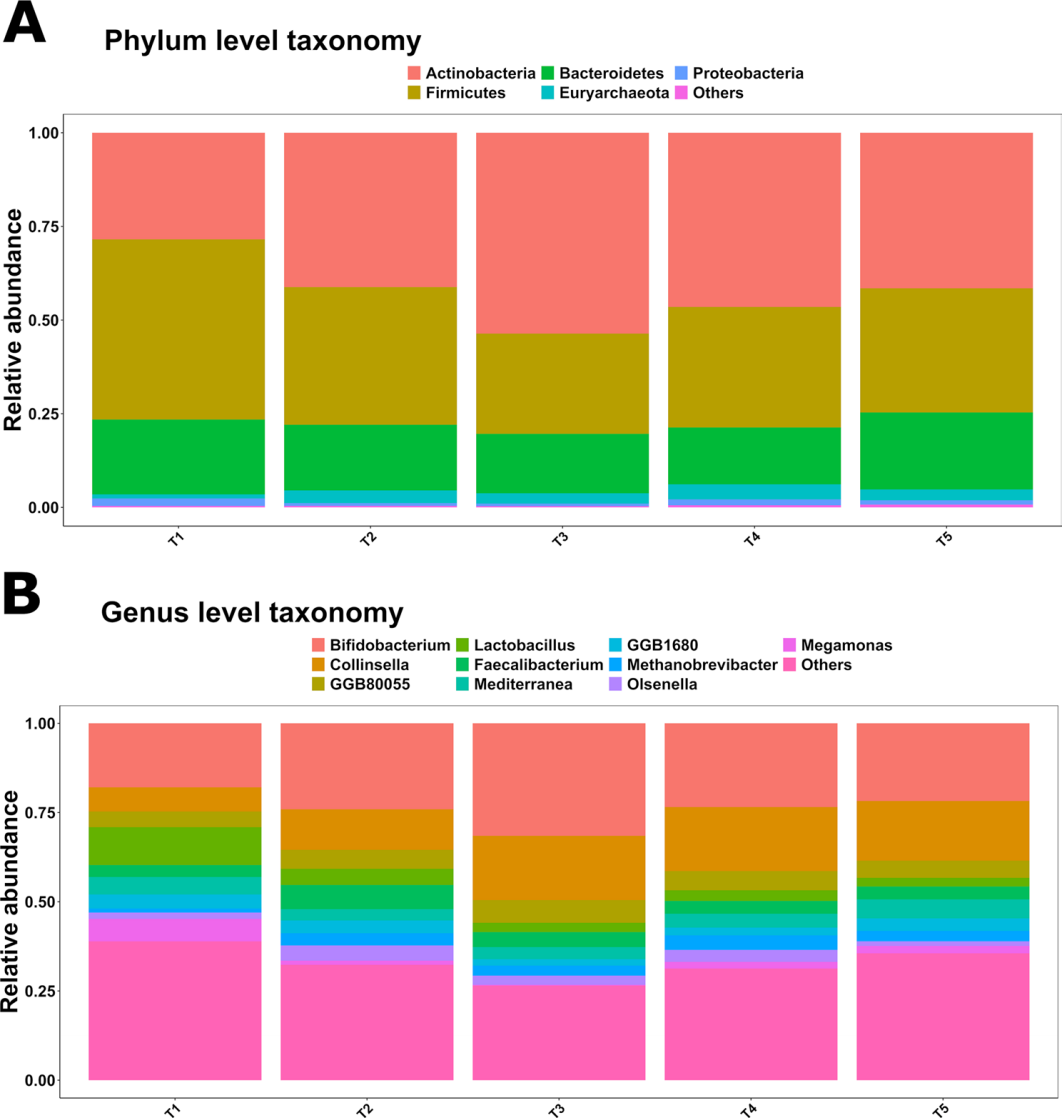
Relative abundance at phylum level (**A**), and genus level (**B**), across the treatments. T1: 0 mg/kg; T2: 150 mg/kg; T3: 300 mg/kg; T4: 450 mg/kg; T5: 600 mg/kg (*n* = 12 cages per treatment)

At phylum level (Fig. 5A), cumulatively, *Actinobacteria* was the most abundant (42.24% ± 13.86%), followed by *Firmicutes* (35.38% ± 11.81%), *Bacteroidetes* (17.84% ± 6.71%), *Euryarchaeota* (2.83% ± 1.63%) and *Proteobacteria* (1.23% ± 1.76%), which explained the 99.5% of the phylum-level bacterial diversity. On the other hand, at treatment level, *Actinobacteria* was significantly enriched in T2 to T5 (41.18% ± 12.4%, 53.59% ± 9.87%, 46.48% ± 12% and, 41.52% ± 11.51%) compared to T1 (28.44% ± 11.51%; *P* < 0.05, *Q* < 0.05), *Firmicutes* decreased in T2 to T5 (36.74% ± 10.13%, 26.76% ± 9.02%, 32.17% ± 7.14%, 33.13% ± 6.85%) compared to T1 (48.08% ± 13.81%; *P* < 0.05, *Q* < 0.05) and *Euryarchaeota* was increased in T2 to T5 (3.39% ± 1.55%, 2.75% ± 1.27%, 4.01% ± 1.7%, 2.9% ± 1.31%) compared to T1 (1.1% ± 0.7%, *P* < 0.05, *Q* < 0.05).

**Fig. 6:**
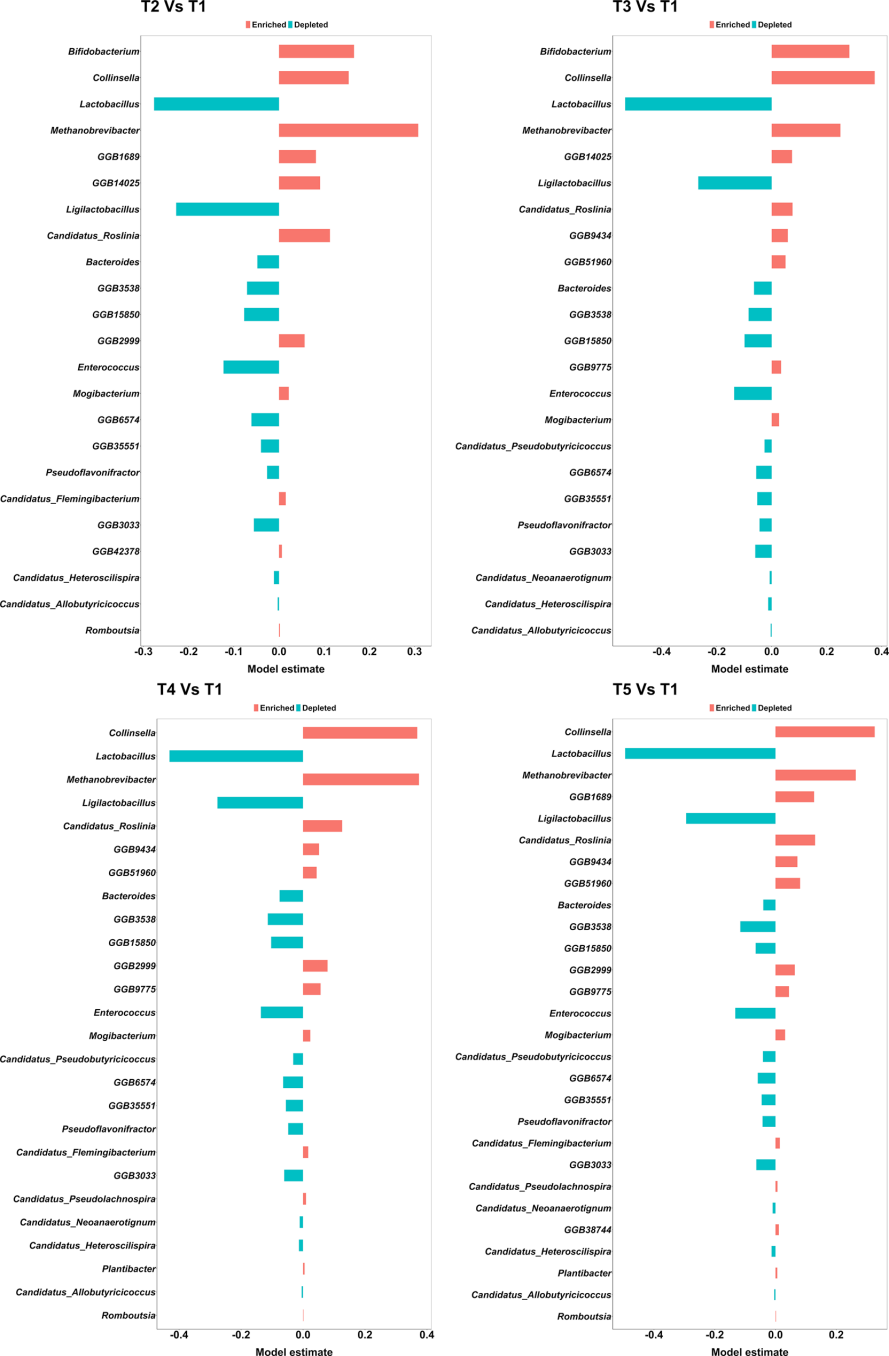
Genera significantly enriched or depleted across the treatment groups, compared to T1 (0 mg/kg). T1: 0 mg/kg; T2: 150 mg/kg; T3: 300 mg/kg; T4: 450 mg/kg; T5: 600 mg/kg (*n* = 12 cages per treatment)

The full list of the composing genera across the treatment groups can be found in Additional file 2, whereas the 10 most abundant genera (Fig. 5B), cumulatively, were *Bifidobacterium* (23.35% ± 8.49%), *Collinsella* (13.93% ± 7.45%), *Flavobacteriaceae* genus-level genome bin (GGB) 80,055 (5.16% ± 1.76%), *Lactobacillus* (4.58% ± 4.96%), *Faecalibacterium* (4.23% ± 2.65%), *Mediterranea* (4.07% ± 2.59%), *Rikenellaceae* GGB1680 (2.96% ± 2.81%), *Methanobrevibacter* (2.83% ± 1.63%), *Olsenella* (2.6% ± 2.94%) and *Megamonas* (2.29% ± 6%). At treatment level, we found that 17 genera were significantly enriched and 14 were significantly depleted across all treatment groups compared to T1 (*P* < 0.05, *Q* < 0.05). The complete list of significant comparisons can be found in Additional file 3 and depicted in Fig. 5, whereas hereafter we describe the significant changes across the ten most abundant genera. *Bifidobacterium* was significantly enriched in T2 and T3 (23.67% ± 6.46% and 30.96% ± 7.2%) compared to T1(17.77% ± 9.37%; *P* < 0.05, *Q* < 0.05), *Collinsella* was significantly enriched in T2 to T5 (11.16% ± 6.68%, 17.7% ± 6.43%, 17.58% ± 6.96%, 16.47% ± 7.51%) compared to T1 (6.72% ± 2.13%; *P* < 0.05, *Q* < 0.05), *Lactobacillus* was significantly depleted in T2 to T5 (4.47% ± 2.38%, 2.59% ± 4.03%, 2.92% ± 2.16% and 2.41% ± 1.88%) compared to T1 (10.51% ± 7.14%; *P* < 0.05, *Q* < 0.05) and *Methanobrevibacter* was significantly enriched in T2 to T5 (3.39% ± 1.55%, 2.75% ± 1.27%, 4.01% ± 1.7% and 2.9% ± 1.31%) compared to T1 (1.1% ± 0.7%; *P* < 0.05, *Q* < 0.05) (Fig. 6).

### Muramidase led to reduced abundance of genes encoding protein and peptidoglycan metabolism, also associated with improved digestibility

A total of 721,334 unstratified gene families were grouped into 4,074 unstratified KEGG orthologs (KO), with an average cumulative abundance of 470,043.89 copies per million (CPM) ungrouped. Both the KO richness (F_(4,44)_ = 6.82; *P* < 0.001) and the Shannon index (F_(4,55)_ = 7.85; *P* < 0.001) were significantly reduced by the treatment, although we did not find significant differences in terms of richness between T2 and T1. Moreover, we observed a decrease in diversity at higher NR and BWG values; indeed both α-diversity indexes were inversely proportional to NR (F_(1,58)_ = 5.85; *P* < 0.05 for the richness and F_(1,58)_ = 10.26; *P* < 0.01 for the Shannon index) and to the log_10_ transformed BWG (F_(1,58)_ = 4.49; *P* < 0.05 for the richness and F_(1,58)_ = 5.63; *P* < 0.05 for the Shannon index). Finally, whilst the Shannon index showed only a trend towards reduction with increased AME (F_(1,58)_ = 3.89; *P* = 0.053), the richness index was significantly reduced at higher AME values (F_(1,58)_ = 5.16; *P* < 0.05) and DMD (F_(1,58)_ = 4.20; *P* < 0.05). The β-diversity analysis measured through the Jaccard distance revealed significant KO compositional differences between all the treatment groups and T1 (*Q* < 0.05) and between T2 and T3. However, the Bray-Curtis dissimilarity indicated a significant separation for only T3, T4, and T5 from T1 (*Q* < 0.05), with only a trend between T2 and T1 (*Q* = 0.063).

#### Treatment-driven correlations

**Fig. 7:**
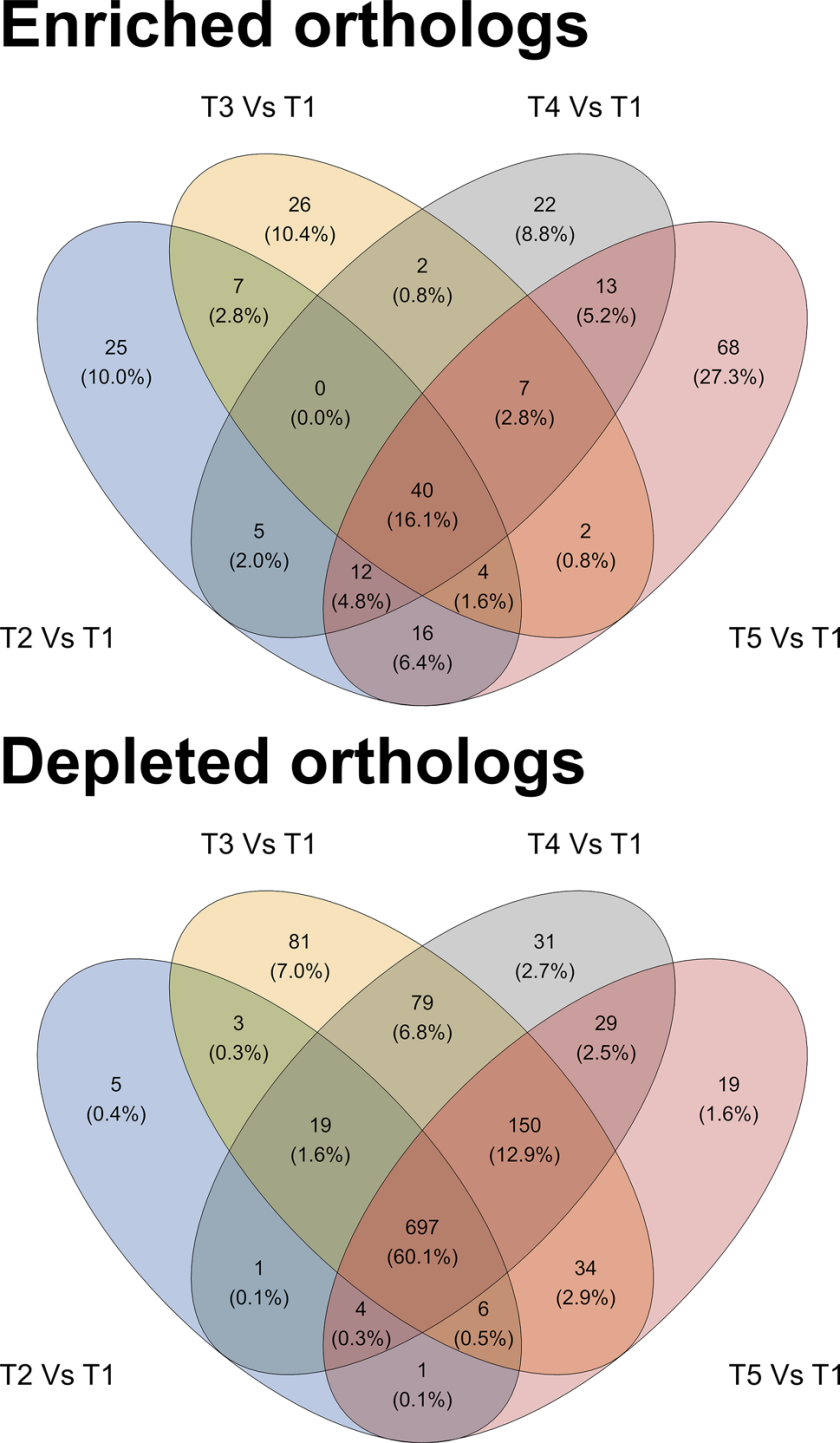
Treatment-driven quantitative effect on the KOs. T1: 0 mg/kg; T2: 150 mg/kg; T3: 300 mg/kg; T4: 450 mg/kg; T5: 600 mg/kg (*n* = 12 cages per treatment)

As depicted in Fig. 7, the abundance of 40 KOs increased across all treatment groups compared to T1. These KOs s primarily encoded for ribosomal proteins (RP-S27e, RP-S28e, RP-L12, RP-L4e, RP-L14e, RP-L30e, RP-L31e, RP-L34e, RP-L37Ae, RP-L39e, RP-L44e) and basal transcription factors (*TBP* and *TFIIB*), together with those encoding carbon (*paaH*, *gap2*, *mvhD*, *mch*, *ACSS1_2*) and methane (*mvhD*, *mch*, *mfnB*, *ACSS1_2*) metabolism. Furthermore, specific KOs s were found to be more abundant in each treatment group, uniquely, when compared to T1, particularly in T5 (68 KOs), where they were mainly encoding for proteins involved in the biosynthesis of cofactors (*AKR1A1*, *ALDH*, *bioB*, *phoA*, *menB*, *pdxB*) and glycosaminoglycan degradation (*hya* and *HEXA_B*). The enrichment analysis of the significantly more abundant KOs in T2, T3 and T4 indicated that carbon (*metF*, *kdgK* and *abfD*), pyrimidine (*pydC* and *ushA*) and cysteine/methionine (*AHCY*, *mtnA*) were enriched in T2, arginine, alanine, aspartate and glutamate metabolism (*argB*, *argG*, *glnA*) and the pentose phosphate pathway (*TALDO1* and *PRPS*) were enriched in T3 and thermogenesis/oxidative phosphorylation (*CYTB*, *ATPeF0A*, *ND1*, *ND2* and *ND6*), the two-component system/ lysine degradation/ aminobenzoate degradation/ butanoate metabolism (*atoD*, *atoA*, *CYTB*, *tctA* and *kdd*) and the metabolism of glycine, serine and threonine (*kbl* and *thrH*) and the degradation of flavonoids (*phy*) were enriched in T5.

**Fig. 8:**
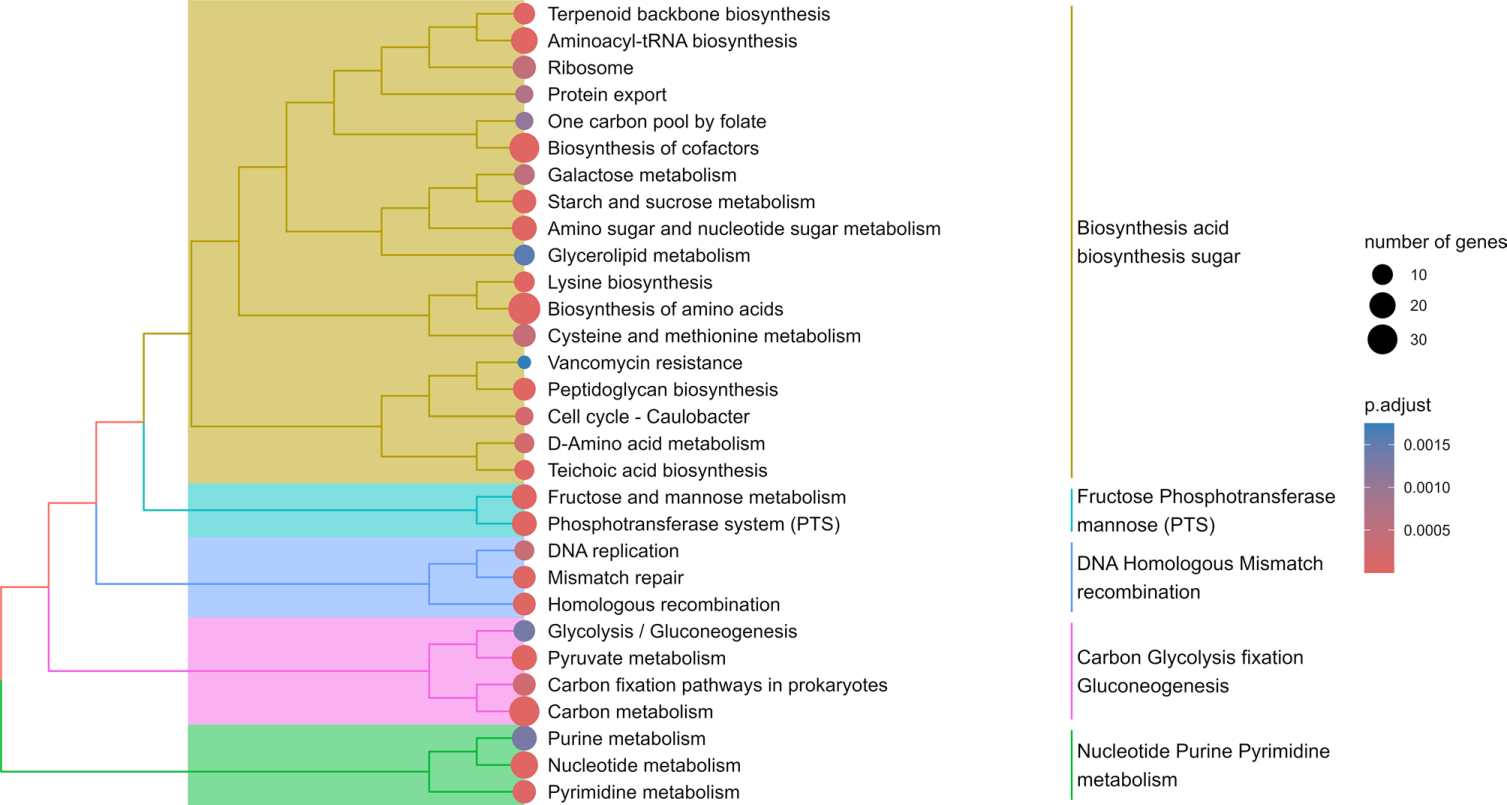
Enrichment tree depicting the down represented functions across all treatments compared to T1. The enrichment tree was built using the enrichment analysis output on the 697 KOs significantly depleted in all treatments compared to T1. T1: 0 mg/kg; T2: 150 mg/kg; T3: 300 mg/kg; T4: 450 mg/kg; T5: 600 mg/kg (*n* = 12 cages per treatment)

A number of KOs were found to be significantly depleted (*Q* < 0.05) in one or more treatments when compared to T1 (Fig. 7). The full list of the significantly differentially abundant KOs can be found in Additional file 3, whereas the enrichment analysis of depleted KOs is discussed hereafter. A total of 697 KOs were significantly less abundant in all the treatments compared to T1, and the biosynthesis of acids and sugars were the main down-represented high-hierarchy functions due to AcM administration (Fig. 8). Specifically, the biosynthesis of amino acids (37 KOs), was significantly down-represented, including cysteine and methionine metabolism (*metA*, *metB*, *metK*, *mtnN*, *thrB*, *cysK*, *patB* and *L-serine dehydratase*), the biosynthesis of lysine (*dapA*, *dapB*, *dapE*, *dapH*, *dapL*, *argD*, *patA* and *lysA*), the metabolism of histidine (*hisA*, *hisB*, *hisE*, *hisH* and *hisI*), glycine, serine and threonine (*glyA*, *thrB*, *thrC* and *L-serine dehydratase*), alanine, aspartate and glutamate (asnA, asnB and argH), the biosynthesis of phenylalanine, tyrosine and tryptophan (*aroD* and *aroE*) and the biosynthesis of arginine (*argD* and *argH*). The metabolism of starch and sucrose (15 KOs), was also reduced in the AcM groups (*PYG*, *sucrose phosphorylase*, *GBE1*, *glgA*, *UGP2*, *glgC*, *malZ*, *INV*, *6-phospho-beta-glucosidase*, *pgmB*, *celA*, *celB*, *scrA*, *bglX* and *inuJ*), together with the depletion of the phosphoenolpyruvate-dependent sugar phosphotransferase system (PTS), and with the biosynthesis of cofactors (31 KOs), including those involved in the metabolism of vitamins, such as thiamine (*adk*, *thiD*, *thiI* and *iscS*), riboflavin (*ribF*), pantothenate (*coaA*, *coaBC* and *coaE*), pyridoxine (*pdxK*) and folate (*DHFR*, *folC*, *folD*, *fhs*, *glyA* and *metK*). Moreover, all the AcM inclusion levels led to a decreased abundance of KOs encoding for the biosynthesis/metabolism of structural cell components, such as teichoic acid (*tagA*, *tagE*, *dltA*, *dltB*, *dltC*, *dltD*, *cpoA*, *mgs* and *bcrC*) and peptidoglycan (*murA*, *murB*, *murC*, *murD*, *murE*, *murF*, *murG*, *bcrC*, *dacC*, *ddl*, *mraY*, *pbp2A* and *pbpB*) of which *murF*, *murG*, *mraY* and *ddl* together with *alr* also led to a decrease in intrinsic vancomycin resistance. Notably, AcM concentration also led to a decreased ability of the caecal microbiota to generate energy through functions such as glycolysis and to a reduction in the metabolism of ribosomal protein and nucleotides, together with a depletion of DNA homologous recombination, replication, and mismatch repair.

#### Phenotypical correlations

A total of 20 KOs were significantly more abundant (*Q* < 0.05) at greater NR values, which were linked to the enrichment of carbon metabolism pathways, including the pyruvate and Tricarboxylic Acid Cycles (TCA cycle), via the KOs *korB*,* fumC* and *MCEE*. Additionally, *rhaD* and *rhaA*, associated with fructose and mannose metabolism, were also enriched as NR increased.

On the other hand, the abundance of 802 KOs was inversely proportional to NR. The enrichment analysis revealed that the biosynthesis of cofactors and amino acids (such as cysteine and methionine metabolism, and alanine, aspartate, and glutamate metabolism), and carbon related pathways (e.g., starch and sucrose metabolism) were down represented in the caeca of birds with improved NR. Furthermore, KOs involved in the biosynthesis of PGN and teichoic acid, such as *murA*,* murB*,* murD*,* murE*,* murF* and *murG*, and*pbp2A* and *pbpB*, *ddl*,* bcrC* and *dacC* were down represented. Finally, KOs associated with propanoate metabolism, protein export and bacterial secretion system, thiamine metabolism, glutathione metabolism, and fatty acid biosynthesis, together with cationic antimicrobial peptide and vancomycin resistance were significantly depleted in chickens with improved NR.

Boh *rfbD* and *rfbB*, which encode for the dTDP-4-dehydrorhamnose reductase and dTDP-glucose 4,6-dehydratase, respectively, showed an inverse relationship with DMD. On the other hand, 9 KOs were more abundant as BWG increased, including F-type H^+^-transporting ATPase subunit a (*ATPeF0A*) and the NADH-ubiquinone oxidoreductase chain 6 (*ND6*), contributing to the thermogenesis. In parallel, 336 KOs were found to be inversely proportional to the BWG, including those encoding for bacterial biosynthesis of cofactors and amino acids (e.g., phenylalanine, tyrosine, tryptophan, histidine, and lysine), carbon and pyruvate metabolism (including glycolysis/gluconeogenesis, galactose metabolism, and carbon fixation), together with the metabolism of nucleotides (including DNA replication and homologous recombination) and the metabolism of starch and sucrose and fructose and mannose. Furthermore, we also found that 9 KOs encoding for molecules involved in the biosynthesis of the PGN (*dacC*, *mrcA*, *murA*, *murB*, *murE*, *murN*, *pbp2B*, *pbp2X*, *vanY*) and 10 encoding for quorum sensing molecules (*livK*, *secA*, *secG*, *secY*, *yidC*, *spo0A*, *agrC*, *ciaR*, *oppA*, *nisR*) were less abundant in birds wither BWG, together with KOs encoding for molecules involved in phosphotransferase system, protein export, glycerolipid metabolism, fatty acid biosynthesis, mismatch repair, vancomycin resistance, cell cycle (*dnab*, *clpx* and *ftsw*).

### Caecal microbial pathways correlated with nitrogen retention

When grouping the gene families, 419 unstratified pathways were bioinformatically integrated, amongst which the abundance of the L-citrulline biosynthesis pathway, through which glutamine and proline are converted to citrulline, was found to be progressively decreased as the DMD increased. Moreover, a total of 18 microbial pathways were less abundant as the NR increased. Of these, 7 pathways were strictly associated with the microbial generation of energy via the degradation of different types of resources and were grouped within the degradation, utilization, and assimilation class. These were (I) the hexitol fermentation to lactate, formate, ethanol and acetate, (II) the Entner-Doudoroff pathway I leading to energy generation through carbohydrate fermentation, (III) the chitin degradation II (Vibrio), (IV) the glycogen degradation I, (V) the superpathway of N-acetylneuraminate degradation, (VI) the stachyose degradation pathways and (VII) the superpathway of glucose and xylose degradation. At species level, the downregulation of the hexitol fermentation to lactate, formate, ethanol and acetate pathway was mainly due to *Lactobacillus salivarius*, with a significant decrease in T2 and T4 compared to T1, whilst the stachyose degradation pathway was down regulated in *L. aviarius*, *L. crispatus*, *L. ingluviei*, *L. johnsonii* and *L. salivarius* in all the treatments compared to T1 and in *L. gallinarum* and *Megamonas hypermegale* in T3 compared to T1. On the other hand, the chitin degradation II (Vibrio) pathway was depleted in *Bacteroides uniformis* in all treatment groups in comparison to T1.

In parallel, the abundance of pathways belonging to the generation of precursors metabolite and energy class was also inversely proportional to the NR. These pathways were (I) the superpathway of glycolysis and the Entner Doudoroff, (II) the pentose phosphate pathway, depleted in *L. ingluviei* in all the treatment groups compared to T1 and in *M. hypermegale* in T3 compared to T1, (III) the isopropanol biosynthesis (engineered) and (IV) the heterolactic fermentation pathways. Finally, 7 pathways grouped within the biosynthesis class were also significantly less abundant with an increase in NR, which were (I) the diacylglycerol biosynthesis III pathway, (II) and (III) the NAD salvage pathways I and V (PNC IV and V cycles), (IV) and (V) the phosphatidylglycerol biosynthesis I and II (plastidic non plastidic) pathways, (VI) the isoprene biosynthesis I pathway and (VII) the methylerythritol phosphate pathway II. The analysis of these pathways, stratified to the different species, revealed that the diacylglycerol biosynthesis III pathway of *L. johnsonii* and *L. salivarius* was significantly less abundant in all the treatment groups compared to T1.

The superpathway of N-acetylneuraminate degradation (unclassified taxonomy) and the hexitol fermentation to lactate, formate, ethanol, and acetate (both from *L. salivarius* and unclassified bacteria) were also found to decrease as the BWG increased. Moreover, within the same class of degradation, utilization, and assimilation, we found that the anaerobic acetylene degradation (from *L. crispatus* and unclassified species), the ethanolamine utilization pathways (from *L. crispatus*, *Flavonifractor* An10, and unclassified species), and the super pathway of purine deoxyribonucleosides degradation (unclassified taxonomy) were also inversely proportional to BWG. Similarly, three caecal microbial pathways belonging to the fermentation class were decreased as the BWG increased. These were the superpathway of fermentation (as found in *Chlamydomonas reinhardtii*) through which pyruvate is converted to acetate, lactate, ethanol, and H_2_, and two pathways converting pyruvate in butanediol, which were the superpathways of (2,3) and (R, R) butanediol biosynthesis (unclassified taxonomy). Finally, the PGN biosynthesis II pathway (as found in *staphylococci*) was also decreased in the caeca of birds with higher BWG.

### Predicted microbial metabolites

The relative abundance of 77 caecal bacterial metabolites was bioinformatically predicted using Melonnpan, based on the gene family (UniRef90) output of HUMAnN 3. Although the Melonnpan pipeline is based on highly trained and validated data sets, and its predictions are based on the actual microbial functional potential observed in the samples, it is important to note that the metabolites described hereafter are predicted (i.e., not derived from targeted/untargeted metabolomic analysis). The following paragraphs omit the appellation “predicted” to enhance readability.

When compared to T1, 11 metabolites were significantly enriched (*Q* < 0.05) across all treatment groups. These metabolites were grouped into lipid-metabolism molecules such as cholestenone, cholesterol, 3 methyladipate pimelate and X2 hydroxymyristic acid, active metabolites and neurotransmitters such as valeric/isovaleric acid, azelate, azelaic acid, C20:4 carnitine, undecanedioic acid, vitamin related metabolites such as the pyridoxamine and nucleotide/nucleoside metabolites such as the deoxyinosine. In parallel, only inosine (purine nucleoside) was significantly depleted across all treatment groups. On the other hand, when comparing the individual treatment groups to the control group (T1), specific patterns emerged. We observed that n-acetylglutamic acid (arginine precursor) was enriched in T2, whereas cytosine, xanthine, and 7-methylguanine (nucleotide metabolism), together with threosphingosine and 3-methylxanthine (active metabolites) where enriched in T3, whilst hydrocinnamic acid (active metabolite) was depleted. Moreover, uracil, n-oleoylethanolamine, asymmetric (ADMA) and symmetric (SDMA) dimethylarginines (protein degradation residues), palmitoylglycerol (active metabolite) and pseudouridine (nucleotide metabolism) were depleted in T4 only, whereas α-muricholic acid (bile acid) was depleted in T5.

When looking at the intersections between two or more groups, thymine was enriched in all the groups apart from T4. Lithocholate (bile acid), urobilin (bilirubin metabolite), n-acetylglutamate (ammonia cycle and arginine biosynthesis) and caproic acid (active metabolite) were enriched in all the groups apart from T3, and both citrulline (arginine precursor) and phenylacetate (amino acid metabolism) were enriched in all the groups apart from T2. On the other hand, dimethyl lysine (amino acid metabolism) was depleted in both T2 and T4, whilst phytosphingosine (lipid metabolism) was depleted in both T4 and T5. A total of 14 metabolites were found to be depleted across the treatment groups apart from T3, compared to T1; these were bile acid metabolites such as cholate and chenodeoxycholate, lipid metabolites such as arachidonic acid, C16:0-LPC, C18:0-SM, docosapentaenoate, adrenic acid and stearoyl ethanolamide, metabolites involved in the metabolism of protein or amino acids, such as imidazole propionate, ADMA and creatine, and active metabolites or neurotransmitters such as the n-acetylputrescine, diacetylspermine, malonate. Finally, thymine (enriched in T2; T3; T5) and deoxyinosine (enriched in all treatments) were positively correlated with NR (*Q* < 0.05).

## Discussion

The superfamily of lysozyme is composed of hydrolytic enzymes targeting the β-1-4 glycosidic bond of the bacterial PGN [50, 51]. Here, we present the findings from our 20-week laying hen (Lohmann Brown) study, where we tested, for the first time, four inclusion levels of AcM on digestibility and microbiota/microbiome.

Phenotypically, we found that the intervention with AcM led to an increased NR and BWG across all treatments whilst also noticing a significant linear relationship between these two variables. This positive correlation, previously reported in literature, is likely to reflect the improved protein retention leading to increased body mass [52]. In parallel, we observed distinct effects on DMD and AME, with significant improvements observed primarily at 300 mg/Kg of AcM (T3) and to a lesser extent with T4 only for AME, while a decrease was noted in T2. Our observations could suggest that an optimal dosage of AcM may be 300 mg/kg, with a possible negative effect at lower concentrations. Previous studies showed that AcM targeted the degradation of free PGN fragments, particularly at duodenal level, thus increasing the surface area available for nutrient absorption [24], which could be compatible with our observations of improved NR. For the sake of completeness, we acknowledge that the current manuscript is focused on exploring microbial mechanisms potentially associated with the digestibility phenotype. Corresponding performance parameters such as laying production data and egg quality are not included herein but have been reported previously [53].

Moreover, AcM also led to the modulation of the caecal microbiota, suggesting a deep structure-function relationship between the host and microbiota due to treatment. The most important change at phylum level was the switch between *Firmicutes* and *Actinobacteria*. Indeed, *Firmicutes* was the most abundant phylum in T1, whilst *Actinobacteria* was the most abundant one in T2 to T5. This change is particularly relevant as although *Firmicutes* is recognized as being composed of fiber fermenting species and usually predominant in the caeca of chickens [54, 55], *Actinobacteria* members have been proposed as probiotic, especially useful in poultry farming due to their role in short chain fatty acid production [56]. In parallel, we found that all levels of AcM inclusion resulted in a decrease in *Lactobacillus* abundance, and a linear mixed model confirmed a significant inverse relationship between *Lactobacillus* log_10_ abundance and NR in our study (F_(1,58)_ = 7.32; *P* < 0.05). Other authors reported that *Lactobacillus* directly competes with the chicken host for amino acids in the small intestine, due to the inability of this genus to synthesize these molecules [57]. Therefore, it is plausible that the reduction of this genus, as observed here, is likely to reflect the improved protein utilization by the host due to the increased NR. This could also support previous observations showing that AcM primarily exerts indirect effects on microbiota, with no or limited direct antibacterial activity on live cells [24]. It ought to be acknowledged that previous evidence indicates that the presence of *Lactobacillus* spp. is positively associated with increased health and performance [58–60]. On the other hand, we previously showed that a reduction of *Lactobacillus* in the foregut of broilers was linked to ameliorated performance [61]. This contrasting evidence indicates that the relationship between this genus and the host is not fully elucidated yet, suggesting that future studies should focus their attention on assessing the role of this genus within the gastrointestinal tract of chickens and linked to different phenotypes beyond performance.

We observed that *Collinsella* had an opposite trend compared to that of *Lactobacillus*, and a previous study had described the same inverse relationship between these two genera [62], which might indicate a possible link between the abundance of these two groups. *Collinsella* has been associated with both positive and negative health effects on different animal species and humans [63]. Importantly, its abundance has been previously positively correlated with the expression of *Claudin-1*, resulting in increased intestinal epithelial barrier function [64]. *Bifidobacterium* has been previously reported as a possible probiotic genus in laying hens, likely correlated with increased performance and egg quality [65]. Interestingly, we reported the enrichment of *Bifidobacterium* only in T2 and T3, which although could be consistent with the phenotype observed in T3, it is somewhat incompatible with the reduced DMD, or AME observed in T2. It ought to be noticed that we observed an increase in methanogens in all the treatments compared to T1, as shown by the enrichment of *Methanobrevibacter.* Although, depending on the absolute genus abundance, *Methanobrevibacter* could be directly related to increased methane emissions, it could also present opportunities for biogas production, following the re-use of excreta in anaerobic digesters [66].

The unstratified analysis of the KOs revealed that 13 genes involved in the biosynthesis of the PGN were significantly down represented in all treatments compared to T1. Amongst these genes, *murA*, *murB*, *murC*, *murD*, *murE*, *murF*, *murG*, *mraY*, *ddl*, encode for 7 essential and 2 redundant proteins leading the synthesis of cytosolic PGN precursors, where MurA, MurB, MurC, MurD and MurE catalyze the very first steps of the process, converting uridine diphosphate N-acetylglucosamine (UDP-GlcNAc) to form UDP-N-acetylmuramic acid pentapeptide [67]. A stratified analysis of these KOs revealed that only a proportion belonged to *Lactobacillus* species, whilst most of the copies were related to other species or unclassified. This could, therefore, suggest that the observed reduction in the ability to synthesize PGN was not directly, or at least in part, linked to a reduced microbial abundance. Indeed, our findings could suggest that AcM may have led to the selection of a functional genetic potential less prone to PGN synthesis, possibly due to the selection of species with less active PGN turnover.

Finally, the analysis of the predicted metabolites revealed that AcM could enhance the production of microbial molecules related to lipid-metabolism, active metabolites and neurotransmitters (valeric/isovaleric acid, azelate, azelaic acid, anandamide C20:4, carnitine and undecanedioic acid), metabolism of vitamins and nucleotides/nucleosides, whilst possibly depleting inosine. Interestingly, valeric acid glycerides, normally produced by microbiota members, have been correlated with increased performance and lower risk associated with necrotic enteritis in broilers [68], whilst L-carnitine has been shown in correlation to increased egg production when administered with omega-3 fatty acid diets [69]. It is important to acknowledge that this analysis is purely predictive, and although based on validated and well established bioinformatic pipelines, it is intended solely to provide insights into the metabolic potential based on the genetic potential explored via the metagenomic analysis.

## Conclusion

Our results support the view that the intervention with AcM is safe for laying hens and enhanced the digestibility of proteins, likely contributing to the observed increase in BWG. These findings suggest that AcM could be an effective tool to optimize protein utilization in laying hens, potentially reducing the need for high-protein diets. Furthermore, our findings could reinforce the previously proven concept that AcM does not hydrolyze PGN of live bacteria, whilst leading to the indirect modulation of the microbiota, driven by the reduced protein availability in the caeca, with some measurable effects on the gene encoding PGN synthesis. Additionally, our observations that certain effects were more pronounced at specific doses of AcM (e.g., 300 mg/Kg) suggest that AcM supplementation may be most effective at optimized concentrations. Overall, these results offer practical implications leading to enhanced gut health in laying hens.

## Supplementary Information

Below is the link to the electronic supplementary material.

Supplementary Material 1: Additional file 1. Ingredient and chemical composition of the control diet

Supplementary Material 2: Additional file 2. Genus level composition across the treatment groups

Supplementary Material 3: Additional file 3. List of differentially abundant genera across the treatment groups, compared to T1 (0mg/kg)

Supplementary Material 4: Additional file 4. List of differentially abundant KOs across the treatment groups, compared to T1 (0mg/kg)

Supplementary Material 5: Additional file 5. Raw data collected during the animal study

## Data Availability

The shotgun metagenomics dataset generated during this study (i.e., NovaSeq X Plus Series paired-end 150 fastq reads) can be found in the European Nucleotide Archive repository at: https://www.ebi.ac.uk/ena, with accession number PRJEB85873.The remaining datasets used and/or analyzed during the current study are included in the manuscript or supporting files.
