# Supplementary material for: Dietary muramidase leads to the downregulation of peptidoglycan biosynthesis and to caecal microbial modulation in laying hens
Source: Anim Microbiome. 2026 Jan 20;8:7. doi: 10.1186/s42523-025-00506-9 (PMC12821794; doi:10.1186/s42523-025-00506-9)
Supplement: Supplementary file 1 — Supplementary Material 1: Additional file 1. Ingredient and chemical composition of the control diet [file 42523_2025_506_MOESM1_ESM.docx]

| **Ingredient and chemical composition of the control diet** | |
| --- | --- |
| **Ingredients** | **(%, or otherwise mentioned)** |
| Barley raw ground | 25.00 |
| Maize raw ground | 25.00 |
| Wheat raw ground | 14.44 |
| Prairie meal | 5.00 |
| Soya Ext hipro | 16.00 |
| Full fat soya Cherwell | 2.00 |
| L Lysine | 0.15 |
| DL Methionine | 0.10 |
| Soya oil | 1.50 |
| Dicalcium Phos. Flour | 0.80 |
| Limestone Trucal 52 | 9.00 |
| Salt | 0.25 |
| Sodium Bicarbonate | 0.15 |
| Layer 1 Premix* | 0.10 |
| Ronozyme Hiphos | 0.0012 |
| Ronozyme Multigrain GT | 0.008 |
| Titanium Dioxide | 0.50 |
| Total | 100 |
|  |  |
|  |  |
|  |  |
| **Calculated Nutrient Content** | |
| Metabolisable energy (Kcal/Kg) | 11.56 |
| Crude protein | 17.48 |
| Crude fat (oil EE) | 3.78 |
| Crude fibre | 2.89 |
| Ash | 12.36 |
| Total lysine % | 0.910 |
| Av. Lysine % | 0.835 |
| Total threonine % | 0.639 |
| Total methionine % | 0.403 |
| Total methionine+cystine % | 0.698 |
| Tryptophan % | 0.187 |
| Calcium % | 3.838 |
| Total phosphorus % | 0.485 |
| Available phosphorus % | 0.282 |
| Analysed Chemical Composition | |
| Dry Matter | 89.87 |
| Crude Protein | 16.58 |
| Oil A | 3.55 |
| Crude fiber | 2.36 |
| Ash | 14.2 |
| * Provided per kg of diet: vitamin A (6000 IU), vitamin D3 (3000 IU), vitamin E (10000 IU), vitamin B12 (25 µg), vitamin B2 ( 0.8 mg), vitamin K (1.01 mg), Nicotinic acid (10 mg), Pantothenic acid (4.018 mg), Folic acid (0.3 mg), Fe (9.9 mg), Mn (80.166 mg), Cu (5 mg), Zn (59.94 mg), I (0.99 mg), Se (0.15 mg). | |
